# Case report: A case of primary cardiac malignant mesothelioma

**DOI:** 10.3389/fonc.2024.1356592

**Published:** 2024-06-17

**Authors:** Ao Wang, Baohui Liu, Shengjun Dong, Yujiu Wang

**Affiliations:** Department of Cardiovascular Surgery, Binzhou Medical University Hospital, Binzhou, Shandong, China

**Keywords:** primary cardiac malignant tumors, mesothelioma, surgical treatment, radiation therapy, case report

## Abstract

Primary cardiac malignant tumors are extremely rare, making up about 10% of all primary cardiac tumors. Most of these tumors are primary sarcomas, with primary mesothelioma being even less common. This report details a 53-year-old male patient diagnosed with primary cardiac malignant mesothelioma. The patient had symptoms of chest pain and difficulty breathing. A CT scan showed an enlarged heart, fluid around the heart, and irregular thickening of the pericardium. Diagnosis was confirmed through a surgical biopsy, which showed the presence of malignant mesothelioma. After the procedure, the patient received appropriate cardiac support. Although stable at discharge, the patient unfortunately died three months later due to severe wheezing. There may be a potential link between exposure to radioactive iodine treatment and this outcome. This case highlights the diagnostic and treatment challenges of primary cardiac malignant tumors and reminds physicians to consider this rare disease when evaluating patients with similar symptoms.

## 1 Introduction

The incidence of primary cardiac tumors is extremely low, with most being benign and only 10% classified as malignant (1, 2). Among the malignant cases, angiosarcoma and poorly differentiated sarcomas are the most common types, making primary cardiac malignant mesothelioma even rarer. (3–5) (**Table 1**). The discovery of patients often occurs incidentally during imaging examinations conducted for unrelated reasons, resulting in a missed opportunity for timely treatment (10). We present a case report of a 53-year-old male patient diagnosed with primary cardiac malignant mesothelioma, whose symptoms were alleviated following surgical intervention. This report provides comprehensive insights into the diagnosis, treatment, and prognosis of this particular case, aiming to enhance understanding of the disease.

**Table 1:** Four cases of cardiac mesothelioma were previously reported.

Title	Reporting time	Gender	Risk Factors	Main symptoms	Location	Treatment	Prognosis
Cardiac mesothelioma (6)	1991						
Cardiac mesothelioma (7)	1996						
Primary cardiac mesothelioma presenting with fulminant recurrent pericarditis: a case report (8)	2023	male	BNT162b2 COVID-19 vaccine	Recurrent pericardial effusion, chest pain , dyspnea	the entire heart and the great vessels	NSAIDs, colchicine, prednisone, Pericardiocentesis, Resection of the pericardium	The patient passed away three weeks post-surgery
Case report and analysis of the literature on sarcomatous mesothelioma of the left atrium (9)	2023	female		Dyspnea, palpitations, syncopal, signs of global heart failure	Within the left atrium	Cardiac mass resection, mitral valve replacement	The patient succumbed to tumor recurrence in the left atrium 9 months post-surgery

However, the original records for the 1991 and 1996 cases could not be obtained. The remaining patients succumbed to heart mesothelioma within one year after undergoing surgery.

## 2 Case report

The patient, a 53-year-old man, presented with chest tightness and wheezing of unknown cause for one year, which had worsened over the past two days. Physical examination revealed weak breath sounds in the left lung and distant heart sounds. Chest CT showed an enlarged heart, irregular thickening of the pericardium with predominant dirty layer, pericardial effusion, bilateral pleural effusion, and enlarged lymph nodes around the great vessels of the upper mediastinum. Venipuncture examination for tumor markers was negative except for slightly elevated ferritin (403.10 ng/ml). The patient had a history of hyperthyroidism and hyperthyroid heart disease treated with methimazole, propranolol, and two courses of iodine-131 (I-131) radiotherapy. In 2021, he underwent pericardiocentesis twice at another medical facility due to worsening chest tightness and wheezing caused by significant pericardial effusion. Preoperative bedside echocardiography revealed reduced motion in the middle and lower segments of the ventricular septum, left ventricular lateral wall, left ventricular anterior wall, inferior apical segment of the left ventricular wall, and middle segment of the left ventricular posterior wall. However, the remaining segments exhibited acceptable levels of ventricular wall motion amplitude and systolic thickening rate. The ejection fraction was measured at 42%, with a ventricular septum thickness of 8 mm, a left ventricular end-diastolic diameter of 45 mm, and a left ventricular posterior wall thickness of 8 mm. Additionally, a fluid dark area was detected within the pericardial cavity. The end-diastolic width measurements were as follows: 27 mm posterior to the left ventricle, 35 mm lateral to the left ventricle, 14 mm anterior to the right ventricle, 16 mm lateral to the right atrium, and 39 mm for both the right atrium and inferior apex regions. Doppler ultrasound indicated mild regurgitation in both the aortic and bicuspid valves along with minimal tricuspid valve regurgitation.

**Figure 1:**
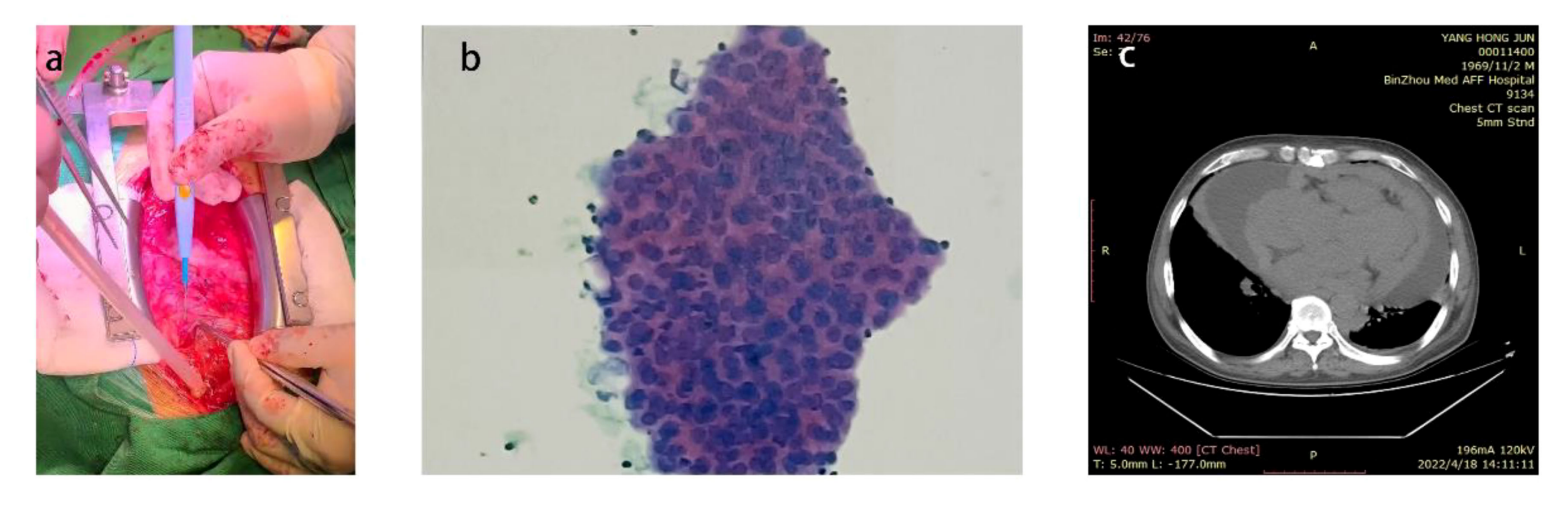
**(A)** The patient presented with a significant volume of pericardial effusion within the pericardial cavity. **(B)** Pathological results of pericardial effusion **(C)** Preoperative chest CT prompted a large number of pericardial effusion.

**Figure 2:**
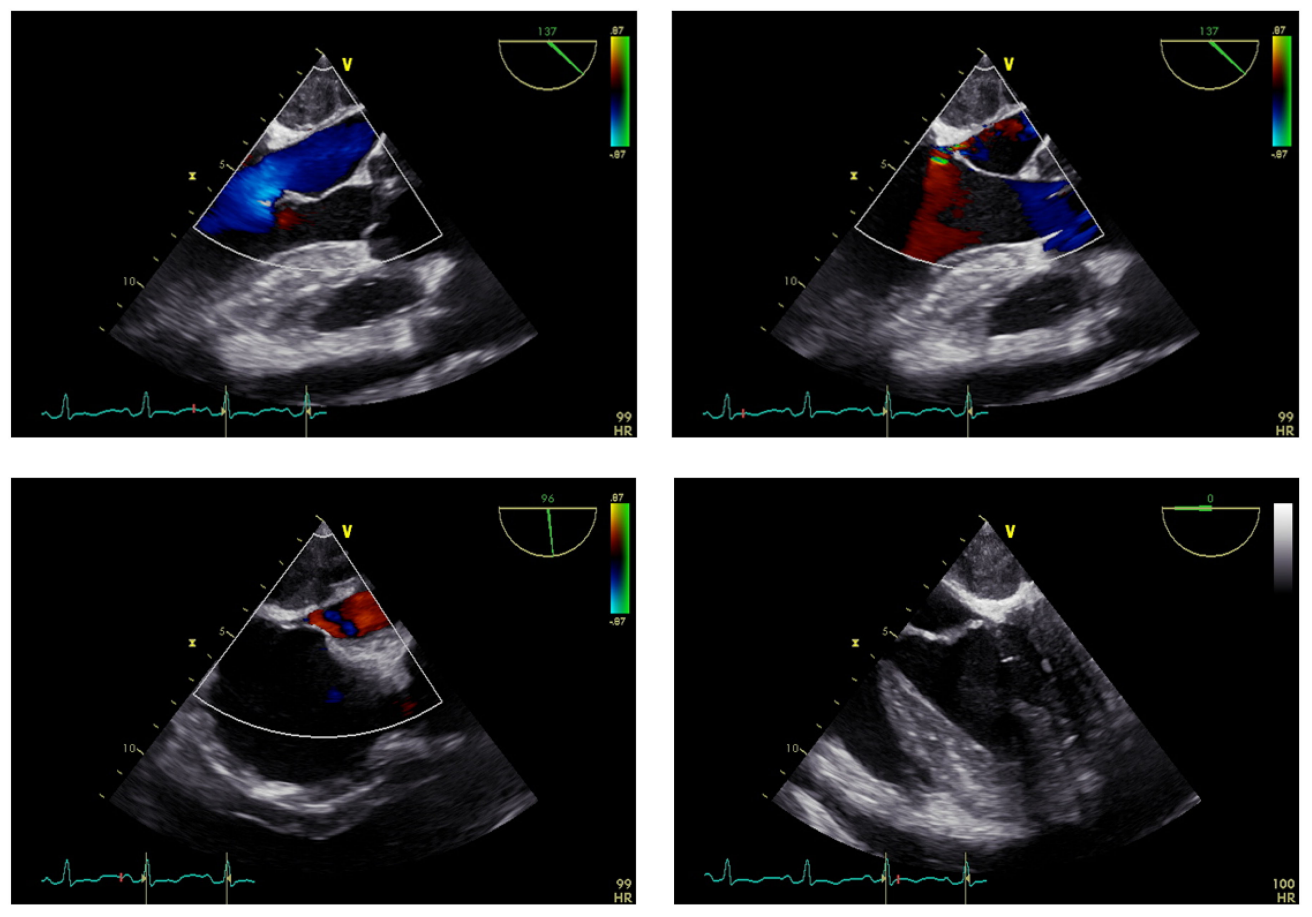
Preoperative TEE examination results.

**Figure 3:**
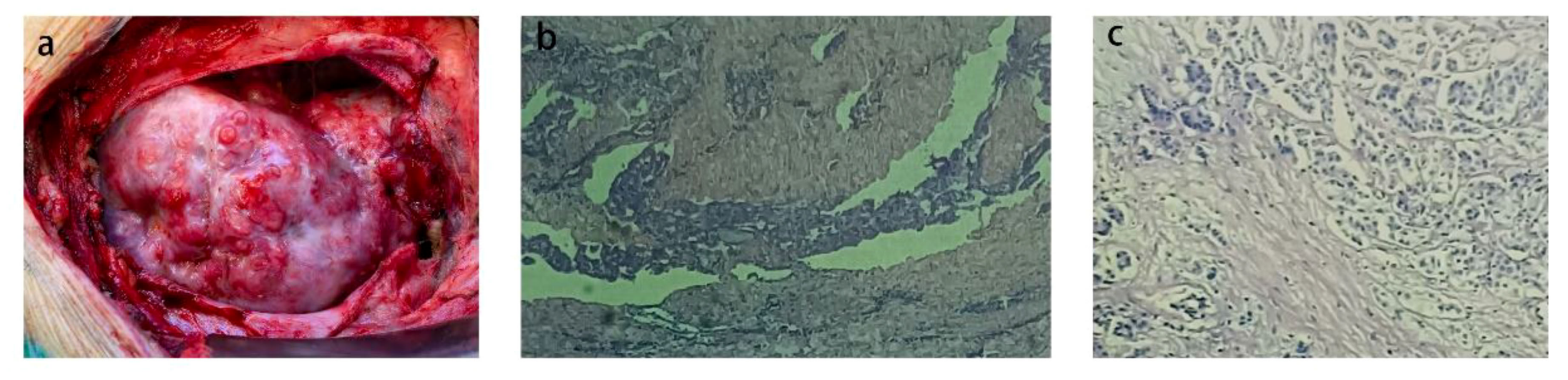
**(A)** Photographs of the heart during surgery **(B)** The pericardium pathological results **(C)** Pathological results of cardiac mass.

After the patient’s thyroid function improved, he underwent surgical treatment at our hospital, specifically a thoracotomy and cardiac tumor biopsy procedure (**Figure 1**). The diagnosis was reconfirmed through a repeated preoperative transesophageal echocardiogram (TEE) (**Figure 2**). During the chest incision, increased tension was observed in the pericardial cavity. A pericardial window was created near the aortic root, revealing an overflow of pale-yellow liquid. We extracted samples of this pericardial effusion to relieve cardiac compression and closely monitored changes in heart rate and blood pressure. In total, approximately 1200 ml of pale-yellow pericardial effusion was aspirated. The pericardium showed thickening and signs of inflammation, with fibrin deposition and adhesion. Upon separation and exploration, it was discovered that the entire heart, as well as its inlet and outlet, were enveloped by a densely connected cardiac mass adhering to the epicardium. The mass exhibited significant rigidity, resulting in impaired cardiac function (**Figure 3**). After thorough analysis, surgical removal of the tumor was deemed unfeasible. Therefore, only a portion of the cardiac mass and pericardium were excised for pathological examination. To prevent recurrence of tamponade caused by massive pericardial effusion, partial resection of the left pericardium and mediastinal pleura was performed to establish communication between the pericardial sac and thoracic cavity. Hemostasis was strictly maintained during surgery, with the placement of one drainage tube each in the pericardial sac, mediastinum, and left thoracic cavity before layered closure of the chest wall incision. Intraoperative blood loss totaled 100 ml without necessitating transfusion. The patient’s blood pressure measured 117/73 mmHg, heart rate was 125 beats per minute, and SpO_2_ level was 98%.

Following the surgical procedure, the patient received a comprehensive treatment regimen that included cardiotonic agents, diuretics, vasodilators, heart rate modulation, and supplemental thyroxine. Three days post-operation, bedside echocardiography revealed bilateral chamber enlargement with normal chamber diameter. The interventricular septum and left ventricular free wall thickness were within normal limits, while ventricular wall motion amplitude and thickening of shrinkage rate were observed. The ejection fraction was measured at 56%. Additionally, the interventricular septum thickness was found to be 9 mm, the left ventricular end-diastolic diameter before and after the operation was 46 mm, and the left ventricular posterior wall thickness was 9 mm. Pericardial cavity and liquid dark space measurements showed a width of 9 mm for the left ventricle after diastole, 18 mm for the left ventricle side, 9 mm for the right side, 16 mm below the roof on the right side before diastole, and an apex measurement of 12 mm. Doppler ultrasound indicated mild aortic regurgitation, mild mitral regurgitation, and moderate tricuspid regurgitation. Furthermore, estimated pulmonary artery systolic pressure was recorded at 42 mmHg per month. Despite being eligible for both heart transplantation and tumor-related treatment, the patient preferred to be discharged to recuperate at home. Consequently, on the fifth day after surgery, the patient was discharged. The patient spent 46 hours in the intensive care unit and underwent mechanical ventilation for 43 hours. Their hospital stay lasted a total of 21 days. Upon discharge, the patient exhibited satisfactory recovery from the operation, with a well-healed chest incision showing no signs of redness, swelling, or exudation. The patient’s condition remained stable without fever, cough, or sputum, and they were able to engage in mild activities without discomfort. The physical examination at discharge revealed strong heart sounds and a consistent rhythm. The patient was instructed to take digoxin tablets, furosemide tablets, spironolactone tablets, sustained-release potassium chloride tablets, bisoprolol fumarate tablets, and levothyroxine sodium tablets upon discharge. During follow-up, the patient experienced persistent wheezing symptoms two weeks after being discharged but relied solely on oral medication and home rest without specialized intervention. Unfortunately, the patient succumbed to his condition three months post-discharge.

Analysis of tumor markers in the pericardial effusion revealed significant elevations in CA19–9 (49.85 U/ml), CA125 (75.28 U/ml), non-small cell carcinoma (451.30 ng/ml), squamous cell carcinoma-associated antigen (17.71 ng/ml), and carbohydrate antigen 50 (35.10 IU/ml). Additionally, there were notable increases in CA15–3 (75.28 U/ml) but no elevation in CA72–4 (0.88 U/ml), neuron-specific enolase (8.57 ng/ml), or CA-242 (1.08 IU/ml). The pathology of the pericardial effusion revealed the presence of tumor cells in both the smear and sediment. Pathological examination of the cardiac mass demonstrated diffuse proliferation of epithelioid cells, exhibiting non-specificity, nucleoli, mitotic figures, adenoid structure, and solid nest arrangement of cells. Immunohistochemical staining showed positive expression for CK+, CR+, Vimentin+, focal positivity for CK7+, MC+, and D2–40+; negative expression for CK20-, and weakly positive WT-1+. The Ki-67 proliferation index was approximately 20%, suggesting a tendency towards epithelioid malignant mesothelioma. Histopathological examination of the pericardium revealed hyperplasia of epithelioid cells without atypia, nucleoli, or mitotic figures. Adenoid structure and solid nests were observed within the cell arrangement. Immunohistochemical staining showed positive expression for CK19+ and scattered weak positivity for MC; negative expression for galectin-3-. Additionally, there was positive expression for CK7+ but negative expression for CK20- and Villin-. These findings further support a diagnosis consistent with epithelioid malignant mesothelioma.

## 3 Discussion

Mesothelioma is a rare cancer originating from mesothelial cells, primarily affecting the pleura, peritoneum, and pericardium. The main subtypes of mesothelioma include epithelioid mesothelioma, sarcomatoid mesothelioma, and biphasic mesothelioma (11). Each subtype exhibits distinct pathological features, clinical manifestations, and prognoses. Epithelioid mesothelioma is the most common subtype, characterized by tumor cells with epithelioid features. It grows slowly and generally has a favorable prognosis. Sarcomatoid mesothelioma, on the other hand, displays sarcomatoid characteristics with rapid growth and aggressive invasion, leading to a poor prognosis. The primary treatments include chemotherapy or radiotherapy, while immunotherapy is less effective compared to epithelioid mesothelioma. Biphasic mesothelioma comprises both cell types mentioned above and has an intermediate prognosis between the two subtypes. The treatment approach primarily involves comprehensive management with surgery, chemotherapy, and radiotherapy; however, further exploration is needed for immunotherapy (11–13).

Primary cardiac malignant tumors are exceedingly rare, and primary cardiac malignant mesothelioma is even more uncommon. Consequently, a lack of understanding of this condition may result in misdiagnosis and delayed treatment. Additionally, pericardial effusion frequently accompanies cardiac malignancies. Tumors in the atria or atrioventricular valve region may present with symptoms that resemble mitral or tricuspid valve stenosis (3). Tumor infiltration into the myocardium can cause symptoms characteristic of restrictive cardiomyopathy, primarily manifesting as heart failure (14). These findings are consistent with the current case. In May 2020, our hospital’s endocrinology clinic observed that patients presenting with chest tightness and chest pain may have been experiencing symptoms of heart disease. Cardiac color Doppler ultrasound revealed left ventricular hypertrophy, reduced left ventricular wall motion, decreased left ventricular diastolic function, mild tricuspid regurgitation, pericardial effusion, and an ejection fraction of 56%. At this time, the patient had received one dose of iodine-131. From 2021 to 2022, the patient’s symptoms of chest tightness and asthma worsened along with orthopnea. Subsequent cardiac color Doppler ultrasounds also showed a significant amount of pericardial effusion. Despite multiple visits to various hospitals during this period (with diagnoses including hyperthyroidism, arrhythmia, pericardial effusion, and pericarditis), no cardiac tumor was identified. It is evident that primary cardiac malignant mesothelioma can be easily misdiagnosed and lead to delayed treatment due to its rarity and lack of specific clinical symptoms.

The role of CT in diagnosing various primary malignant tumors is crucial, serving as an important adjunct. It enables visualization of tumor invasion and provides information on the extent of tumor attachment (15). However, in this case, the patient’s chest CT scan revealed only cardiac enlargement, pericardial effusion, and irregular thickening of the visceral pericardium, without definitive evidence of neoplastic disease. The detection of tumor markers in venous serum lacks specificity for common identification; however, there is a clear association between pericardial effusion and tumor marker detection. Therefore, it can be inferred that relying solely on imaging and serum tumor marker detection may not completely exclude the possibility of primary cardiac malignant mesothelioma, especially if the patient presents with recurrent pericardial effusion, cardiac enlargement, and pericardial thickening.

The patient exhibits significant risk factors for cardiac malignancy. It remains unclear whether there is a history of I-131 radiation therapy. While it is evident that radiation therapy can cause side effects, such as cardiac structural alterations and various cardiovascular disorders, there are few reports and studies on radiation-induced heart disease, including I-131 therapy-related cardiac tumors (16). No other potential exposures have been identified, and no occupational exposures associated with the patient’s career as a truck driver have been found.

Current treatment modalities for cardiac malignant tumors include surgical intervention, radiotherapy, and chemotherapy. The postoperative survival rate is closely associated with the completeness of surgical resection (17). In this case, the tumor extensively involved the entire heart, including its entrances and exits, and adhered closely to the epicardium. Due to the difficulty of surgical resection, only palliative treatment was administered, resulting in a grim prognosis for the patient.

## Data availability statement

The raw data supporting the conclusions of this article will be made available by the authors, without undue reservation.

## Ethics statement

Written informed consent was obtained from the individual(s) for the publication of any potentially identifiable images or data included in this article.

## Author contributions

AW: Writing – original draft, Writing – review & editing. BL: Investigation, Writing – review & editing. SD: Investigation, Writing – review & editing. YW: Investigation, Writing – review & editing.
